# Comparison of Laparoscopic Sleeve Gastrectomy (LSG) with Laparoscopic Gastric Bypass (LRYGB) in Bariatric Surgery

**DOI:** 10.7759/cureus.14022

**Published:** 2021-03-21

**Authors:** Manzar Ali, Safdar Ali Khan, Muhammad Mushtaq, Syed Aftab Haider

**Affiliations:** 1 General and Colorectal Surgery, Ibn-e-Siena Hospital and Research Institute, Multan, PAK; 2 General Surgery, Ibn-e-Siena Hospital and Research Institute, Multan, PAK; 3 Anesthesia, Nishtar Medical University Hospital, Multan, PAK

**Keywords:** bariatric surgery, laparoscopic roux-en-y gastric bypass, laparoscopic sleeve gastrectomy

## Abstract

Introduction

The objective of our study was to compare the 30-day post-operative outcomes of laparoscopic sleeve gastrectomy (LSG) and laparoscopic Roux-en-Y gastric bypass (LRYGB).

Materials and Methods

This randomized controlled study contained patients who underwent bariatric surgery from June 13, 2018 to October 12, 2019. A total of 116 patients having body mass index (BMI) > 35 kg/m^2 ^(morbidly obese), age 18 to 65 years and with failure of conservative treatment were included. Group I patients underwent bariatric surgery using LRYGB technique while group II patients underwent bariatric surgery using the LSG technique. All patients were followed 30 days after surgery to determine early post-operative outcomes.

Results

The demographic profile, such as age and BMI, was similar between the groups. The mean operating time was 72 ±12 minutes in the LSG group and 156 ± 18 min in the LRYGB group (p-value 0.0001). Major complications were seen in five (8.62%) patients in LSG patients and in four (6.89%) patients in LRYGB group (p-value 0.12). Minor complications were seen in 21 (36.2%) patients in LSG group and in 19 (32.75%) patients in LRYGB group (p-value 0.15). The length of stay in the hospital in LSG group was 6.2±4.2 versus 9.4± 4.6 (p-value 0.0002).

Conclusion

Both LRYGB and LSG are effective and safe bariatric procedures with a similar incidence of major complications. However, LSG is associated with shorter operative time and hospital stay. Long-term follow-up studies are required to compare the effectiveness of these procedures.

## Introduction

Obesity is one of the most challenging chronic diseases in the western world as well as in Pakistan [1]. Half of the people in this world consider themselves either overweight or obese. According to WHO (2016) statement, 13% of the world’s adult population is obese, 39% of adults above 18 years are overweight. The worldwide prevalence of obesity nearly tripled between 1975 to 2016 [2]. As per WHO, 26% of Pakistani women and 19% of Pakistani men are obese [3]. Pakistan is a country, where awareness of diet is different compared to the rest of the world. Usually, the diet contains energy-dense with saturated fats, high sugar, and high trans-fatty acids [4].

Obesity is challenging to control by medical therapy and drug treatment. The most effective way to treat obesity is bariatric surgery [5]. The foremost goal of operating bariatric surgery is to decrease obesity-associated comorbidities, such as diabetic mellitus and dyslipidemia. Long-term results show that bariatric surgery is the only effective management for obesity [5].

Laparoscopic Roux-en-Y gastric bypass (LRYGB) is a “gold standard” bariatric surgical procedure. LRYGB surgery provides two surgical modifications: (i) it restricts the gastric capacity and (ii) it diverts the swallowed nutrients away from the proximal part of the small intestine [6]. The dramatically increasing prevalence of obesity in the population warrants durable treatment options, such as bariatric procedures, a necessity.

Recently there is much attention gained by laparoscopic sleeve gastrectomy (LSG), which was described by Regan et al. [7]. LSG is restrictive surgery with no mal-absorptive effect and it preserves the integrity of the pylorus and avoids the intestinal bypass. LSG is a stapled gastroplasty with the protection of natural anatomy but the permanent subtraction of a portion of the stomach. LSG is associated with low risk, excellent short-term efficacy, a lesser amount of invasiveness, and lower complications [8]. Hence, the majority of laparoscopic surgeons prefer LSG as a stand-alone bariatric procedure [9,10], even though both types of bariatric procedures are commonly performed all over Pakistan. The potential conclusion of the present study to make a generous impact on the selection of bariatric surgery. Therefore, we decided to conduct this comparative study to compare the early post-op complications of LSG with the LRYGB technique in patients undergoing bariatric surgery.

## Materials and methods

This randomized controlled trial (NCT04779723) containing 116 patients who underwent bariatric surgery was conducted from June 13, 2018 to October 12, 2019. Inclusion criteria were patients of age 18 to 65 years, body mass index (BMI) kg/m^2^ (morbidly obese), with failure of conservative treatment. The exclusion criteria were severe indicative gastroesophageal reflux disease (GERD), conversion of another bariatric procedure, large hiatal hernia, and patients with inflammatory bowel disease.

Approval of the study was taken from the research evaluation unit of the hospital. All patients were informed about the risks and benefits of both operations, and a written informed consent taken from individual patients before including them in the study and by insuring them the confidentiality of their data and identity.

**Figure 1:**
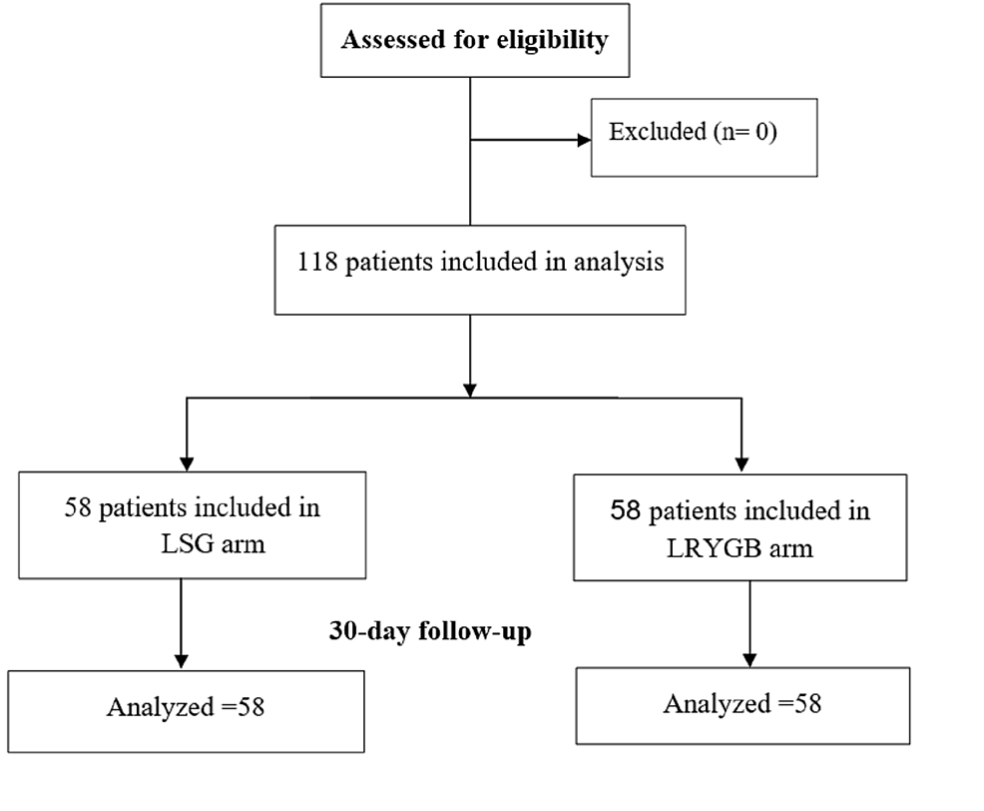
Flow diagram (study overview).

All morbidly obese patients were distributed into two groups using the computer-generated random number table. Fifty-eight patients allotted for the LSG group and another 58 patients to the LRYGB group (Figure 1).

LRYGB technique was performed by placing four to six trocars, a 150-cm ante-colic Roux-limb gastric pouch (30 to 50 ml) was created with linear stapled or circular stapled gastro-jejunostomy based on the surgeon preference, A 50-cm long biliopancreatic limb was chosen. Passive drainage was kept near to the gastro-jejunostomy. On the fourth post-operative day, the drainage was removed. For LSG, 35 Fr bougie was used for the calibration of a gastric tube. A 3 to 6 cm of longitudinal incision of the stomach was done at pylorus to the angle of His. After completing the resection, the staple line was sewn using absorbable suture. The abovementioned standardized procedures were done in all patients.

Data analysis was carried out by SPSS v23.0 (IBM Inc., USA), quantitative variables were compared between the LSG and LRYGB group using an independent sample t-test. For qualitative variables, frequency (%) was calculated and a comparison between the groups was made using Chi-square test. Statistical p-value < 0.05 indicated as significant.

## Results

Mean age was 46.6 ±12.3 years in the LSG group and 43.4 ±11.2 years in the LRYGB group (p-value 0.14). Mean BMI was 46.2 ± 4.2 kg/m^2^ versus 47.3 ±4.6 kg/m^2^ in LSG and LRYGB, respectively (p-value 0.18). There were 22 (37.98%) hypertensive, 32 (55.1%) diabetic, and 39 (67.2%) dyslipidemia patients in LSG group versus 26 (44.28%) hypertensive, 28 (48.27%) diabetic, and 42 (72.41%) dyslipidemia patients in LRYGB group. Detailed data are presented in Table 1.

**Table 1:** Baseline characteristics. N, number of patients; SD, standard deviation.

Description	LSG group (N= 58)	LRYGB group (N= 58)	P-value
Age (mean ± SD)	46.6 ± 12.3	43.4 ± 11.2	0.14
Male/female (%)	15/43 (25%/75%)	19/39 (33%/67%)	0.40
BMI (mean ± SD)	46.2 ± 4.2	47.3 ± 4.6	0.18
Hypertension (%)	22 (37.9%)	26 (44.28%)	0.45
Diabetes mellitus (%)	32 (55.1%)	28 (48.27%)	0.44
Dyslipidemia (%)	39 (67.2%)	42 (72.41%)	0.54

The mean operative time was 72 ± 12 min in LSG and 156 ± 18 min in LRYGB group (p-value 0.0001). The length of stay in LSG group was 6.2 ± 4.2 days versus 9.4 ± 4.6 days in LRYGB group (p-value 0.0002). Major complications were diagnosed five (8.62%) patients in LSG patients and in four (6.89%) patients in LRYGB group (p-value 0.12). Leakage was diagnosed in three (5.16%) patients in LSG group and in one (1.72%) patient in LRYGB group (p-value 0.31), bleeding was diagnosed in one (1.72%) patient in LSG group versus one (1.72%) patient in LRYGB group (p-value 1.0). Wound infections were diagnosed in one (1.72%) patient in LSG group and in two (3.44%) patient in LRYGB group (p-value 0.56). There was no operative mortality. Minor complications were diagnosed in 21 (36.2%) patients in LSG group and in 19 (32.75%) patients in LRYGB group (p-value 0.15). Hair loss and nausea were commonest complications, with an insignificant difference between the groups. Detailed perioperative and post-operative data are shown in Table 2.

**Table 2:** Comparison of Peri and post-Operative morbidity.

	LSG group ( n= 58)	LRYGB group (n= 58)	p-value
Procedure duration (min)	72 ± 12	156 ± 18	0.0001
Hospital stay (days)	6.2 ± 4.2	9.4 ± 4.6	0.0002
Major complications (%)	5 (8.62%)	4 (6.89%)	0.12
Leak (%)	3 (5.16%)	1 (1.72%)	0.31
Bleeding (%)	1 (1.72%)	1 (1.72%)	1.0
Wound infections (%)	1 (1.72%)	2 (3.44%)	0.56
Mortality (%)	0.0	0.0	--
Minor complications (%)	21 (36.2%)	19 (32.75%)	0.15
Anemia (%)	3 (5.16%)	4 (6.88%)	0.69
Hair loss (%)	8 (13.79%)	9 (15.51%)	0.79
Nausea (%)	8 (13.79%)	6 (10.34%)	0.57

## Discussion

The LSG and LRYGB are the commonest techniques of performing bariatric surgery. These procedures are safe and effectively reduces the weight and comorbidities in long-term follow-up [11,12]. Recent studies have described using LSG as a stand-alone procedure for morbidly obese patients. The result of these studies is similar to gastric bypass regarding metabolic effects [13,14].

In the present study, we found that LSG is safer than LRYGB as it has a statistically short operating procedure time (p-value 0.0001) and hospital stay (p-value 0.0002). We also found an overall lower rate of complication in the LSG group but it was non-significant in both groups.

Studies conducted on the short-term outcome of the LRYGB and LSG, also reported higher operative time and long hospital stay using the LRYGB technique, but with insignificant difference [15]. Li et al. recently reported that the complication rate is higher in LRYGB technique as compared to LSG technique (6.9% vs 0.9%, p < 0.001) [16]. Peterli et al. reported that less than 30 days post-operative complications are higher in LRYGB than in LSG without statistically significant (17.2% vs 8.4%, p-value 0.067) [17]. Moreover, a prospective study conducted by Leyba et al. did not report any major complication between the two techniques [18]. Vidal et al. showed a significant difference in blood transfusion requirements: 8.8% in LRYGB vs 1.7% in LSG group (p-value 0.015) [19].

Our results are almost similar to the FINISH trial, which exclusively studied the post-operative complications between LSG and LRYGB groups [20]. The trial reported lower procedural time and complications in the LSG group; 13.2% versus 26.5% in LRYGB group. The trial reported that LSG is superior to LRYG regarding early post-operative outcomes. Another study reported that complications of LSG are minimal; around 1% to 3% in regular cases if adopted as a first-line procedure and can go up to 16% in repeated procedures [21].

Another study conducted by the national surgical quality improvement program (NSQIP) reported that risk adjustment for LSG is 1.32 times lesser than LRYGB [22]. Even though they had a lower complication rate in the LSG group, LSG was associated with a higher incidence of leakage due to long-staple lines as compared to the LRYGB technique.

A meta-analysis study conducted on 30-day complications comparison between LSG and LRYGB by Kumar et al. concluded that LSG had lower odds of leak and death than LRYGB and is safe regarding the short-term outcomes [23].

Limitations of the study

The major limitation of the present study is the small sample size and we followed the patients only for 30 days. There is a need to conduct large-scale studies from high volume centres to further validate the gold standard technique for bariatric surgery.

## Conclusions

Both LRYGB and LSG are effective and safe bariatric procedures with a similar incidence of major complications. However, LSG is associated with shorter operative time and hospital stay. Long-term follow-up studies are required to compare the effectiveness of these procedures. Based on the present study results, we suggest to prefer LSG as a preferred bariatric procedure.

## References

[1] Gallagher EJ, LeRoith D, Karnieli E (2008). The metabolic syndrome—from insulin resistance to obesity and diabetes. Endocrinol Metab Clin North Am.

[2] Chooi YC, Ding C, Magkos F (2019). The epidemiology of obesity. Metabolism.

[3] Siddiqui M, Hameed R, Nadeem M (2018). Obesity in Pakistan; current and future perceptions. J Curr Trends Biomed Eng Biosci.

[4] World Health Organization (2014). Global status report on noncommunicable diseases 2014. https://www.who.int/nmh/publications/ncd-status-report-2014/en/.

[5] Adams TD, Davidson LE, Litwin SE (2012). Health benefits of gastric bypass surgery after 6 years. J Am Med Assoc.

[6] Suter M, Donadini A, Romy S, Demartines N, Giusti V (2011). Laparoscopic Roux-en-Y gastric bypass: significant long-term weight loss, improvement of obesity-related comorbidities and quality of life. Ann Surg.

[7] Regan J, Inabnet W, Gagner M, Pomp A (2003). Early experience with two-stage laparoscopic Roux-en-Y gastric bypass as an alternative in the super-super obese patient. Obes Surg.

[8] Brethauer SA, Hammel JP, Schauer PR (2009). Systematic review of sleeve gastrectomy as staging and primary bariatric procedure. Surg Obes Relat Dis.

[9] Gagner M, Hutchinson C, Rosenthal R (2016). Fifth International Consensus Conference: current status of sleeve gastrectomy. Surg Obes Relat Dis.

[10] Rondelli F, Bugiantella W, Vedovati MC (2017). Laparoscopic gastric bypass versus laparoscopic sleeve gastrectomy: a retrospective multicenter comparison between early and long-term post-operative outcomes. Int J Surg.

[11] Fobi MA, Lee H, Holness R, Cabinda D (1998). Gastric bypass operation for obesity. World J Surg.

[12] Esposito K, Maiorino MI, Petrizzo M, Bellastella G, Giugliano D (2015). Remission of type 2 diabetes: is bariatric surgery ready for prime time?. Endocrine.

[13] Mognol P, Chosidow D, Marmuse JP (2005). Laparoscopic sleeve gastrectomy as an initial bariatric operation for high-risk patients: initial results in 10 patients. Obes Surg.

[14] Almogy G, Crookes PF, Anthone GJ (2004). Longitudinal gastrectomy as a treatment for the high-risk super-obese patient. Obes Surg.

[15] Zak Y, Petrusa E, Gee DW (2016). Laparoscopic Roux-en-Y gastric bypass patients have an increased lifetime risk of repeat operations when compared to laparoscopic sleeve gastrectomy patients. Surg Endosc.

[16] Li K, Gao F, Xue H (2014). Comparative study on laparoscopic sleeve gastrectomy and laparoscopic gastric bypass for treatment of morbid obesity patients. Hepatogastroenterology.

[17] Peterli R, Borbély Y, Kern B (2013). Early results of the Swiss Multicentre Bypass or Sleeve Study (SM-BOSS): a prospective randomized trial comparing laparoscopic sleeve gastrectomy and Roux-en-Y gastric bypass. Ann Surg.

[18] Leyba JL, Llopis SN, Aulestia SN (2014). Laparoscopic Roux-en-Y gastric bypass versus laparoscopic sleeve gastrectomy for the treatment of morbid obesity. A prospective study with 5 years of follow-up. Obes Surg.

[19] Vidal P, Ramón JM, Goday A (2013). Laparoscopic gastric bypass versus laparoscopic sleeve gastrectomy as a definitive surgical procedure for morbid obesity. Mid-term results. Obes Surg.

[20] Helmiö M, Victorzon M, Ovaska J (2012). SLEEVEPASS: a randomized prospective multicenter study comparing laparoscopic sleeve gastrectomy and gastric bypass in the treatment of morbid obesity: preliminary results. Surg Endosc.

[21] Gagniere J, Slim K, Launay-Savary M-V, Raspado O, Flamein R, Chipponi J (2011). Previous gastric banding increases morbidity and gastric leaks after laparoscopic sleeve gastrectomy for obesity. J Visc Surg.

[22] Young MT, Gebhart A, Phelan MJ, Nguyen NT (2015). Use and outcomes of laparoscopic sleeve gastrectomy vs laparoscopic gastric bypass: analysis of the American College of Surgeons NSQIP. J Am Coll Surg.

[23] Kumar SB, Hamilton BC, Wood SG, Rogers SJ, Carter JT, Lin MY (2018). Is laparoscopic sleeve gastrectomy safer than laparoscopic gastric bypass? a comparison of 30-day complications using the MBSAQIP data registry. Surg Obes Relat Dis.

